# Klotho in Osx^+^-mesenchymal progenitors exerts pro-osteogenic and anti-inflammatory effects during mandibular alveolar bone formation and repair

**DOI:** 10.1038/s41392-022-00957-5

**Published:** 2022-05-11

**Authors:** Yi Fan, Chen Cui, Clifford J. Rosen, Tadatoshi Sato, Ruoshi Xu, Peiran Li, Xi Wei, Ruiye Bi, Quan Yuan, Chenchen Zhou

**Affiliations:** 1 State Key Laboratory of Oral Diseases, National Clinical Research Center for Oral Diseases, Department of Cariology and Endodontics, West China Hospital of Stomatology, Sichuan University, 610041 Chengdu, Sichuan China; 2 Hospital of Stomatology, Guanghua School of Stomatology, Sun Yat-Sen University, Guangdong Provincial Key Laboratory of Stomatology, 510055 Guangzhou, Guangdong China; 3 Maine Medical Center Research Institute, Scarborough, ME 04074 USA; 4 Endocrine Unit, Massachusetts General Hospital, Harvard Medical School, Boston, MA 02215 USA; 5 State Key Laboratory of Oral Diseases, National Clinical Research Center for Oral Diseases, Department of Orthognathic and TMJ Surgery, West China Hospital of Stomatology, Sichuan University, 610041 Chengdu, Sichuan China; 6 State Key Laboratory of Oral Diseases, National Clinical Research Center for Oral Diseases, Department of Oral Implantology, West China Hospital of Stomatology, Sichuan University, 610041 Chengdu, Sichuan China; 7 State Key Laboratory of Oral Diseases, National Clinical Research Center for Oral Diseases, Department of Orthodontics, West China Hospital of Stomatology, Sichuan University, 610041 Chengdu, Sichuan China

**Keywords:** Bone remodelling, Differentiation, Endocrine system and metabolic diseases, Inflammation

## Abstract

Maxillofacial bone defects are commonly seen in clinical practice. A clearer understanding of the regulatory network directing maxillofacial bone formation will promote the development of novel therapeutic approaches for bone regeneration. The fibroblast growth factor (FGF) signalling pathway is critical for the development of maxillofacial bone. Klotho, a type I transmembrane protein, is an important components of FGF receptor complexes. Recent studies have reported the presence of Klotho expression in bone. However, the role of Klotho in cranioskeletal development and repair remains unknown. Here, we use a genetic strategy to report that deletion of Klotho in Osx-positive mesenchymal progenitors leads to a significant reduction in osteogenesis under physiological and pathological conditions. Klotho-deficient mensenchymal progenitors also suppress osteoclastogenesis in vitro and in vivo. Under conditions of inflammation and trauma-induced bone loss, we find that Klotho exerts an inhibitory function on inflammation-induced TNFR signaling by attenuating Rankl expression. More importantly, we show for the first time that Klotho is present in human alveolar bone, with a distinct expression pattern under both normal and pathological conditions. In summary, our results identify the mechanism whereby Klotho expressed in Osx^+^-mensenchymal progenitors controls osteoblast differentiation and osteoclastogenesis during mandibular alveolar bone formation and repair. Klotho-mediated signaling is an important component of alveolar bone remodeling and regeneration. It may also be a target for future therapeutics.

## Introduction

Maxillofacial bone has crucial physiological functions, such as formation of the facial framework, protection of digestive and respiratory systems, and providing support for adjacent soft tissues and dental structures.^1^ Bone defects in the maxillofacial region are often seen in clinical practice. The most common causes include inflammation, trauma, oral cancer, and congenital birth defects.^2^ Treatment of defects and regeneration of bone has always been challenging due to its unique features, the aesthetic requirements of the facial skeleton and the significant physical changes that can lead to psychosocial distress.^3^ There are obvious differences between maxillofacial and most other bones due to their distinctive embryonic origin, bone formation, and structure.^4^ Therefore, elucidating the mechanisms of maxillofacial skeletal development and repair is essential to enable innovation in therapeutic strategies for treatment of maxillofacial bone defects.

The fibroblast growth factor (FGF) signalling pathway is essential in maxillofacial bone development. Human gene muations in FGF receptors have been identified as causes of multiple abnormalities in the growth and development of the craniofacial skeleton.^5^ Klotho (KL) is a type I transmembrane protein that is an essential component of FGF receptor 1 (FGFR1) complexes.^6^ The principal role of membrane-anchored Klotho is to act as a co-receptor for fibroblast growth factor 23 (FGF23) as it increases the binding capability of FGFR1 to FGF23. FGF23 is a hormone that is secreted primarily from osteocytes and osteoblasts and serves to maintain mineral ion homeostasis.^7^ Klotho is predominantly expressed in the kidney, parathyroid gland, and choroid plexus.^8^ Recent studies have identified Klotho transcripts in bone, implying a significant local regulatory function of Klotho in bone metabolism.^9^ Klotho-hypomorphic mice have low bone mass and reduced bone turnover.^6^ However, it has been difficult to distinguish whether this phenotype was due to a cell-autonomous functional defect of Klotho in bone or systemic changes due to hyperphosphatemia and hypervitaminosis D. Another study used *Prx1Cre* to conditionally ablate Klotho in mesenchymal stem cells. *Prx1Cre;KL*^*fl/fl*^ mice revealed a skeletal phenotype comparable to controls; but these mice could not increase FGF23 expression in the skeleton under uremic conditions.^10^ Since the discovery of Klotho in osteocytes,^9^ Komaba et al. demonstrated that osteocyte-specific Klotho ablation resulted in a striking increase in bone formation coupled with enhanced osteoblast activity.^11^ The discrepancy in skeletal phenotype of these mouse models was possibly due to a distinct role of Klotho in different populations of bone cells.

Due to the specific embryonic lineages and developmental pattern, most of the craniofacial skeleton originates from neural crest cells and is formed by intramembranous and endochondral ossification, while the limb skeleton is the product of lateral plate mesodermal cells and undergoes endochondral bone formation.^12^ Therefore, the function of Klotho may vary by regions of the skeleton. It is notable that patients with genetic diseases such as tumoral calcinosis, which is associated with missense mutation in Klotho, suffer severe craniofacial abnormalties,^13^ underscoring the essential role of Klotho in craniofacial bone development. However, the regulatory function of Klotho in maxillofacial bone ossification as well as in the pathogenesis of maxillofacial bone loss and repair remains unknown.

In this study, we generated a novel mouse model with a targeted ablation of Klotho in Osterix^+^ (Osx^+^)-mesenchymal progenitor cells to study these questions. Inactivation of Klotho in Osx^+^-progenitors caused skeletal deformities, including decreased mandibular alveolar bone formation and decreased bone resorption. This led to aberrantly high alveolar bone volume during development. Furthermore, we have discovered the regulatory mechanisms used by mesenchymal progenitor-expressed Klotho to affect osteoclastogenesis. We found that in response to alveolar bone loss and traumatic injury, Klotho has a critical role in promoting osteogenesis during bone repair while inhibiting inflammatory-induced bone resorption by targeting tumor necrosis factor signaling and Rankl expression. Moreover, we reported for the first time the presence of Klotho in human alveolar bone and its distinct expression pattern under both healthy and pathological conditions.

## Results

### Targeted ablation of Klotho in Osx^+^-mensenchymal progenitors leads to increased alveolar bone volume

**Fig. 1:**
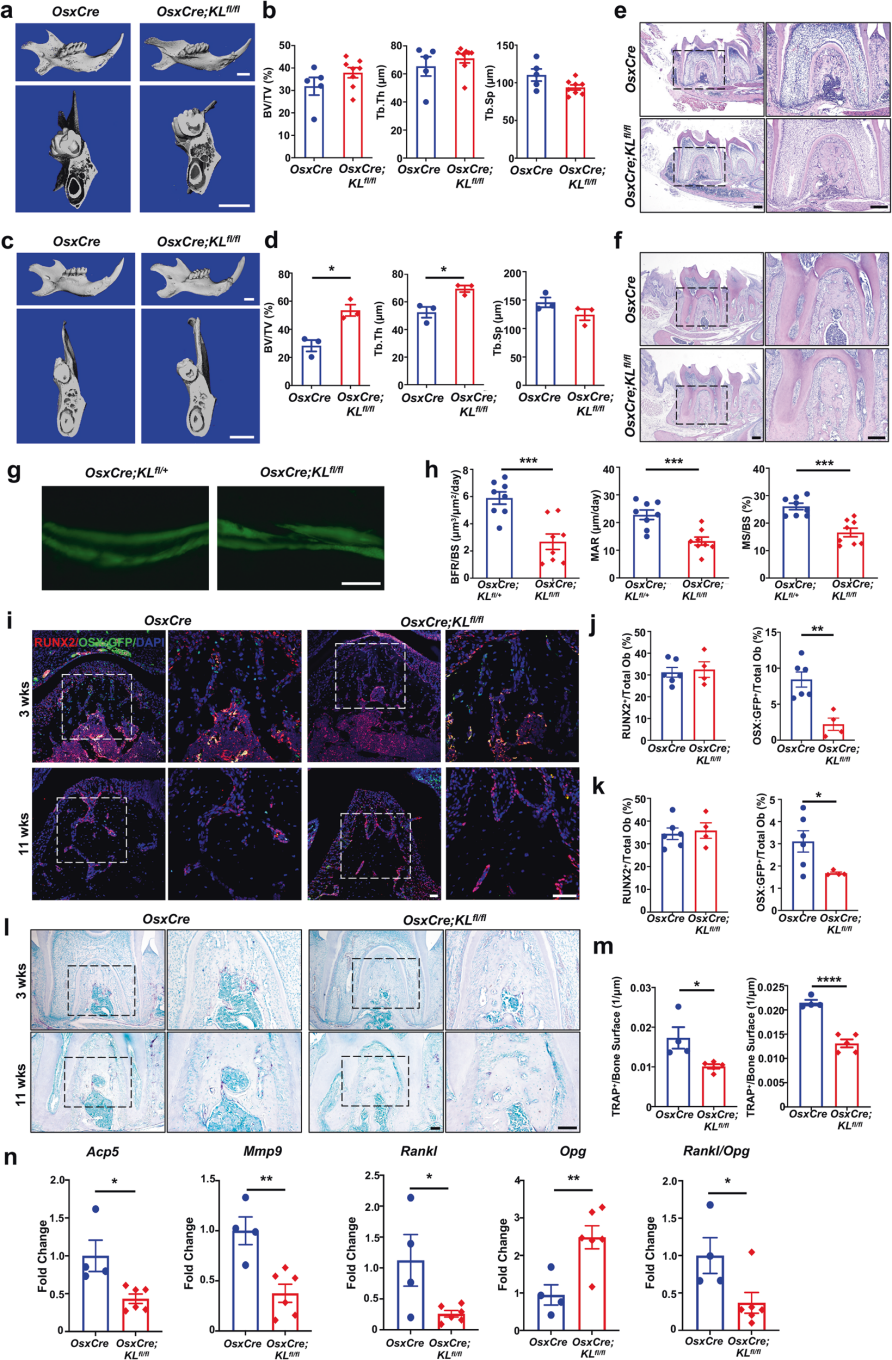
Klotho deficiency in Osx^+^-cells causes impaired bone remodeling. **a, c** Three-dimensional reconstruction from micro-computed tomography scans of the alveolar bone of *OsxCre;KL*^*fl/fl*^ mice and their control littermates at 3 (**a**) and 11 weeks old (**c**). **b, d** Quantitative analyses of bone volume/tissue volume (BV/TV, %), trabecular thickness (Tb.Th, μm), and trabecular separation (Tb.Sp, μm) in 3- (**b**, *n* = 5 in *OsxCre* group and *n* = 8 in *OsxCre;KL*^*fl/fl*^ group) and 11-week-old mice (**d**, *n* = 3). **e, f** Hematoxylin and eosin (H&E) staining of alveolar bone at root bifurcation of mandibular 1st molar in 3- (**e**) and 11-week-old mice (**f**). Higher magnifications of boxed areas show increased alveolar bone mass in *OsxCre;KL*^*fl/fl*^ mice. *n* = 5. **g, h** Calcein double labeling of alveolar bone from control and *OsxCre;KL*^*fl/fl*^ mice. Representative image (**g**) and quantitative analyses of bone formation rate/bone surface (BFR/BS, μm^3^/μm^2^/day), mineral apposition rate (MAR, μm/day) and mineralizing surface/bone surface (MS/BS, %) (**h**). *n* = 8. **i** Double fluorescence staining of RUNX2 and OSX:GFP. Higher magnifications of boxed areas show RUNX2 and OSX:GFP-positive cell distribution at 1st molar root bifurcation. **j, k** Quantification of RUNX2 or OSX:GFP-positive cells/total osteoblasts in 3- (**j**) and 11-week-old mice (**k**). *n* = 6 in *OsxCre* group and *n* = 4 in *OsxCre;KL*^*fl/fl*^ group. **l** TRAP staining. Higher magnifications of boxed areas show the number of TRAP-positive cells decreased in *OsxCre;KL*^*fl/fl*^ mice. **m** Quantification of TRAP-positive cells/bone surface in 3- (left) and 11-week-old mice (right). *n* = 4 in OsxCre group and *n* = 5 in *OsxCre;KL*^*fl/fl*^ group. **n** qRT-PCR analysis of *Acp5, Mmp9, Rankl, Opg*, and *Rankl/Opg* transcription in 11-week-old mice. *n* = 4 in *OsxCre* group and *n* = 6 in *OsxCre;KL*^*fl/fl*^ group. *p < 0.05, **p < 0.01, ***p < 0.001, ****p < 0.0001. All data are shown as the mean ± SEM. Scale bar, 1 mm (**a, c**), 200 μm (**e**), 50 μm (**g, i**), and 100 μm (**l**)

We investigated a possible role for Klotho in maxillofacial bone development by conditionally ablating Klotho expression in Osx-expressing mesenchymal progenitors. This was accomplished by crossing *KL*^*fl/fl*^ mice with *OsxCre* mice (supplementary Fig. 1a, b). *OsxCre;KL*^*fl/fl*^ mice were born with the expected rate of Mendelian inheritance and had similar body weight compared to control littermates (supplementary Fig. 1c). Serum Ca^2+^, Pi, and intact-FGF23 levels were not altered in *OsxCre;KL*^*fl/fl*^ mice, allowing us to isolate the tissue-specific function of Klotho (supplementary Fig. 1e). Alizarin red/Alcian blue staining did not detect significant changes of skeletal mineralization in craniofacial bone of neonatal *OsxCre;KL*^*fl/fl*^ mice (supplementary Fig. 1f). At 3 weeks postnatal, μCT analysis revealed comparable alveolar bone volume in the furcation area of the mandibular first molars in *OsxCre;KL*^*fl/fl*^ mice and controls (Fig. 1a). Mutants had slightly but not significantly increased bone volume/tissue volume (BV/TV, %, *p* = 0.180) and a decrease in trabecular separation (Tb.Sp, μm, *p* = 0.051) (Fig. 1b). We did observe significantly higher alveolar bone volume in these conditional knockout mice at a later stage compared to littermate controls (Fig. 1c). μCT quantitative analysis revealed significantly higher BV/TV (*p* = 0.012) and trabecular thickness (Tb.Th, mm, *p* = 0.020) in the mutants when compared to control littermates. There was no difference observed in Tb.Sp (*p* = 0.170) (Fig. 1d). We also performed H&E staining of mandibular bone from 3- and 11-week-old mice. Not surprisingly, the trabecular bone mass in the furcation area was higher in *OsxCre;KL*^*fl/fl*^ mice. No changes were observed in the histology of mandibular molars (Fig. 1e, f).

### Increased alveolar bone mass in *OsxCre;KL*^*fl/fl*^ is associated with impaired bone remodeling

The increased alveolar bone volume in the Klotho-ablated mice could result from an imbalance of bone formation and resorption. Therefore, we performed dynamic histomorphometry on the alveolar bone. The results showed that mineral surface/bone surface (MS/BS), mineral apposition rate (MAR), and bone formation rate/bone surface (BFR/BS) were markedly downregulated in *OsxCre;KL*^*fl/fl*^ mice compared to controls (Fig. 1g, h). Next, we determined the activities of osteoblast lineage cells by immunofluorescent staining. While the number of Runt-related transcription factor 2 (Runx2)-positive cells were unchanged in mutant mice, Klotho deletion reduced Osx immunoreactivity significantly (Fig. 1i-k). In general Runx2 defines the progenitor cells committed to preosteoblast, while Osx expression signifies the differentiation to an osteoblast.^14^ These results suggest that Klotho ablation primarily affected osteoblast activity and maturation.

Tartrate-resistant acid phosphatase (TRAP) staining revealed significantly decreased TRAP-positive osteoclasts/bone surface in the alveolar bone of *OsxCre;KL*^*fl/fl*^ mice compared to age-matched controls (Fig. 1l, m). In addition, genes related to osteoclast differentiation and activation, such as *Acp5* (*Trap*) and *matrix metalloproteinase 9* (*Mmp9*), were markedly suppressed in mutants. We next assessed the key factors secreted by osteoblasts to mediate bone resorption and found reduced *Receptor activator of nuclear factor-κ B ligand* (*Rankl*), increased *Osteoprotegerin (Opg)* expressions and lower *Rankl/Opg* ratio in the alveolar bone, implying a Klotho mesenchymal progenitor-dependent effect on osteoclastogenesis (Fig. 1n).

### Klotho enhances skeletal system development by promoting the differentiation of Osx^+^-mesenchymal progenitors

**Fig. 2:**
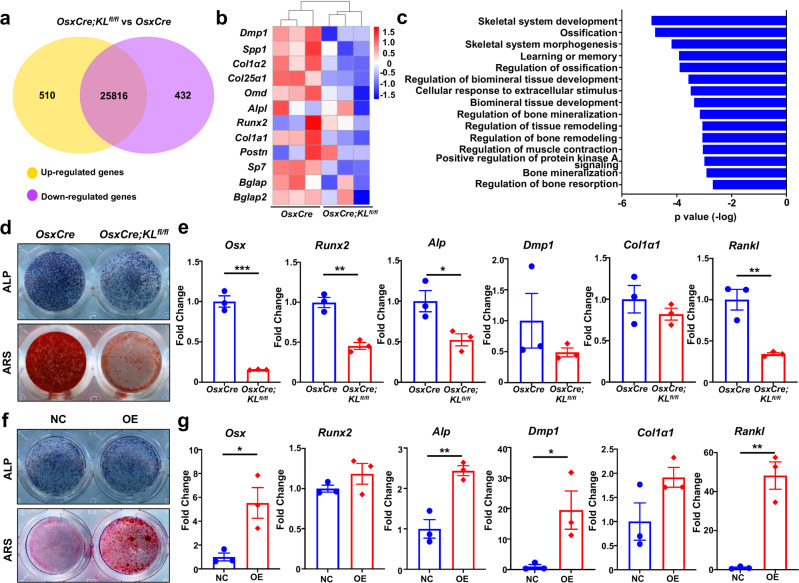
Klotho favors skeletal system development by promoting osteogenic differentiation. **a** Venn diagram showing 510 genes uniquely upregulated while 432 genes downregulated in Klotho-ablated mandibles (*p*-value < 0.05). *n* = 3. **b** Heatmap of representative genes associated with osteogenesis. Red indicates upregulated expression; blue indicates downregulated expression. **c** Gene ontology (GO) enrichment analysis of downregulated genes in total differentially expressed genes (DEGs). **d** Alkaline phosphatase (ALP) staining and alizarin red staining (ARS), respectively, at 7 days (7d) and 14 days of osteogenic induction in primary calvarial osteoblasts from *OsxCre* and *OsxCre;KL*^*fl/fl*^ mice. *n* = 3. **e** Transcription of *Osx, Runx2, Alp, Dmp1, Col1α1*, and *Rankl* after 14d induction. *n* = 3. **f** ALP staining and ARS after transfected lentivirus particles to overexpress Klotho (OE) or empty load (NC). *n* = 3. **g** Transcription of genes after 14 days of induction. *n* = 3. *p < 0.05, **p < 0.01, ***p < 0.001. All data are shown as the mean ± SEM

We established a pivotal function of Klotho in alveolar bone development in vivo, so we next explored how loss of Klotho inhibited ossification. We isolated total RNA from mandibles of *OsxCre;KL*^*fl/fl*^ and *OsxCre* mice and subjected it to RNA sequencing (RNA-seq). The global transcriptome was significantly altered between control and Klotho-ablated mice. Among a total of 26,758 genes expressed, 510 genes were upregulated while 432 genes were downregulated in Klotho-depleted mandibles (Fig. 2a). The crucial regulators associated with osteogenesis, such as Dentin matrix acidic phosphoprotein 1 (Dmp1), Secreted phosphoprotein 1 (Spp1), Collagen type I alpha 2 (Col1α2), and Osteomodulin (Omd), were found to be significantly suppressed in the mutants (Fig. 2b). Correspondingly, gene ontology (GO) analysis showed that genes that were downregulated by Klotho deficiency were highly expressed during skeletal system development, ossification, biomineral tissue development, etc (Fig. 2c).

We continued our investigation into the role of Klotho from Osx^+^ precursors in mediating osteogenesis in vitro. Osteoblasts from *OsxCre* and *OsxCre;KL*^*fl/fl*^ mice were cultured. After 7 and 14 days of culture under osteogenic condition, Klotho-deleted osteoblasts had significantly decreased alkaline phosphatase (ALP) and alizarin red staining (ARS) intensity, accompanied by downregulated osteogenic-related markers, including *Osx, Runx2, Alkaline phosphatase (Alp), Dmp1*, and *Collagen type I alpha 1* (*Col1α1)*, indicating decreased osteogenic differentiation (Fig. 2d, e). In contrast, osteoblasts from *KL*^*fl/fl*^ were transfected by lentiviral to overexpress Klotho and cultured under osteogenic media. Klotho overexpression leads to markedly increased ALP and ARS intensities, accompanied by higher expression levels of osteogenesis-related markers (Fig. 2f, g). These data imply that Klotho enhances craniofacial skeleton development by accelerating osteogenic differentiation of Osx^+^-cells.

### *OsxCre;KL*^*fl/fl*^ mice exhibit profound periapical lesion due to impaired bone remodeling

**Fig. 3:**
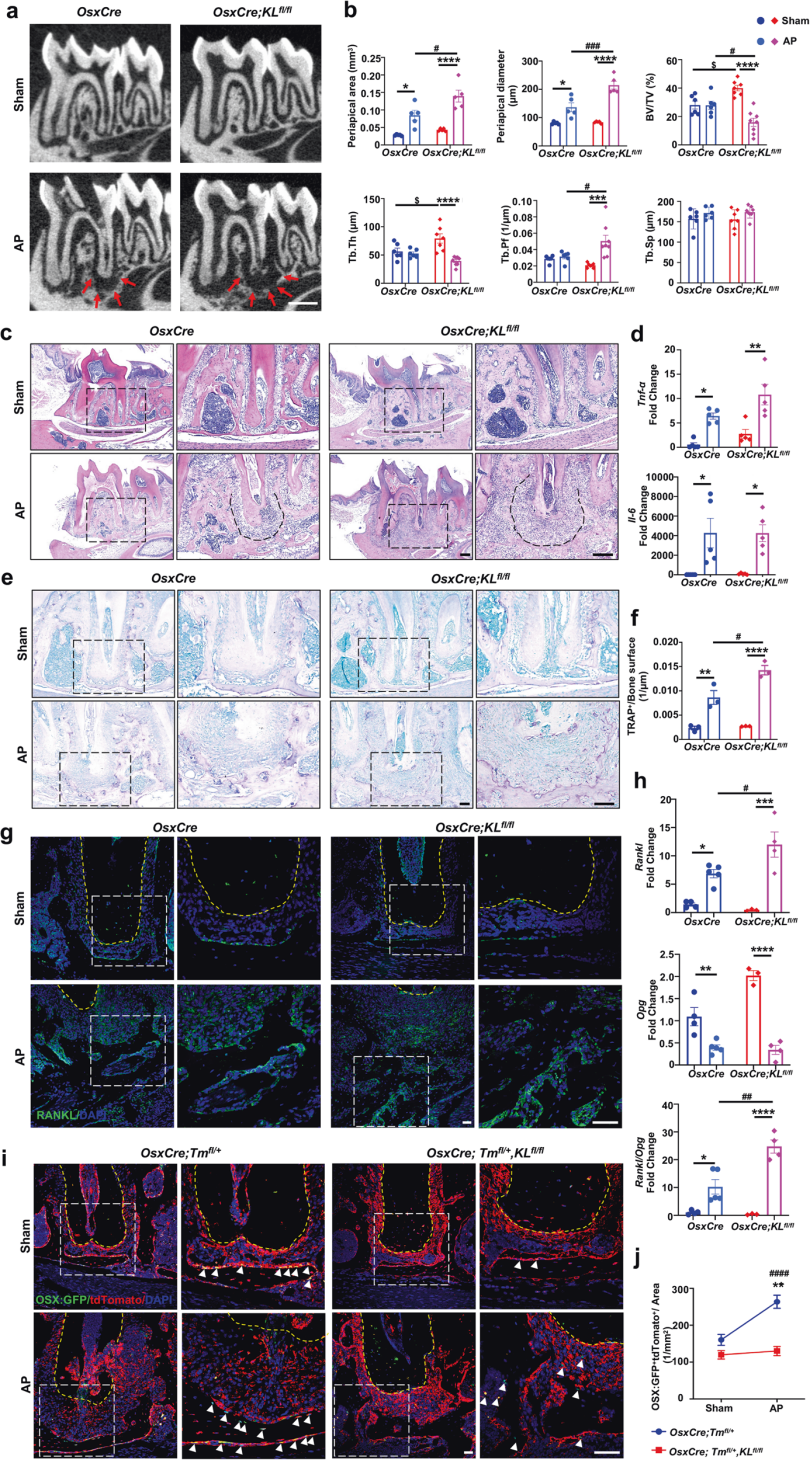
Klotho deletion promotes periapical lesion in alveolar bone. **a** μCT images of the mandibular first molar from *OsxCre;KL*^*fl/fl*^ and control mice in apical periodontitis and sham groups. Red arrow depicts the periapical lesion. **b** Quantitative analysis of periapical lesion area including periapical area (mm^3^) and periapical diameter (μm), *n* = 5; and cancellous bone parameters including BV/TV (%), Tb.Th (μm), trabecular pattern factor (Tb.Pf, 1/μm), and Tb.Sp (μm), *n* = 6 in *OsxCre* Sham and AP group, *n* = 7 in *OsxCre;KL*^*fl/fl*^ Sham group, and *n* = 8 in *OsxCre;KL*^*fl/fl*^ AP group. **c** H&E staining of mandibles depict the periapical lesion induced by apical periodontitis. Dash line shows range of periapical lesion. *n* = 5. **d** Transcription expression of *Tnf-α* and *Il-6* in periapical bone. *n* = 5. **e, f** TRAP staining and quantification of TRAP-positive cells/bone surface. Higher magnifications of boxed areas show more TRAP-positive cells in lesion region of *OsxCre;KL*^*fl/fl*^ mice. *n* = 3. **g** Immunofluorescence staining for RANKL in the periapical region. Boxed areas show higher expression of RANKL in *OsxCre;KL*^*fl/fl*^ AP group. Yellow dash lines depict interface of 1st molar distal root. **h** Gene expression of *Rankl, Opg*, and *Rankl/Opg* ratio in periapical bone of mandibular 1st molar. *n* = 4 in *OsxCre* Sham and *OsxCre;KL*^*fl/fl*^ AP group, *n* = 5 in *OsxCre-*AP group, and *n* = 3 in *OsxCre;KL*^*fl/fl*^ Sham group. **i** Immunofluorescence double staining of OSX:GFP and tdTomato in periapical bone surrounding the distal root of the mandibular 1st molar. Boxed areas show higher magnifications of OSX:GFP and tdTomato double-positive cells in periapical bone. White arrowhead indicates double-positive cells. **j** Quantification of OSX:GFP and tdTomato double-positive cells. *n* = 4 in *OsxCre*-sham group, *n* = 13 in *OsxCre*-AP group, *n* = 8 in *OsxCre;KL*^*fl/fl*^-sham group, and *n* = 12 in *OsxCre;KL*^*fl/fl*^-AP group. *, ^$^, ^#^*p* < 0.05, **, ^##^*p* < 0.01, ***, ^###^*p* < 0.001, ****, ^####^*p* < 0.0001, * indicates AP versus sham, ^$^ indicates *OsxCre* Sham versus *OsxCre;KL*^*fl/fl*^ Sham, ^#^ indicates *OsxCre* AP versus *OsxCre;KL*^*fl/fl*^ AP. All data are shown as the mean ± SEM. Scale bar, 200 μm (**a, c**), 100 μm (**e**), and 50 μm (**g, i**)

The data above indicate that Klotho in osteoblasts is important for mandibular alveolar bone formation. We therefore determined if Klotho deletion would affect pathological bone loss in vivo. We first established a standard apical periodontitis (AP) model. To this end, dental pulp of mandibular first molars in *OsxCre;KL*^*fl/fl*^ and control mice was exposed to the oral microenvironment. μCT revealed that significant alveolar bone loss was found in the mandibular first molar of both *OsxCre;KL*^*fl/fl*^ and control mice 3 weeks after the model induction. Surprisingly, *OsxCre;KL*^*fl/fl*^ AP mice showed a more profound radiolucent area characterized by a significantly increased periapical lesion area and trabecular pattern factor (Tb.Pf, 1/μm) as well as shortened tooth root compared to the control AP group (Fig. 3a, b, supplementary Fig. 2a, b). Histological examination showed that both *OsxCre;KL*^*fl/fl*^ and control mice had preserved periapical structures under normal conditions. AP led to infiltration by inflammatory cells surrounding the apex of affected teeth, accompanied by increased alveolar bone resorption (Fig. 3c). Gene expression analysis demonstrated that AP caused increased expression levels of *Tumor necrosis factor-α (Tnf-α)* and *Interleukin-6* (*Il-6)* compared to the controls (Fig. 3d).

It is noteworthy that mice with Klotho ablation exhibited markedly increased osteoclast number and more severely disrupted dental roots than control mice, suggesting that bone resorption is elevated in inflammatory bone loss in the absence of Klotho (Fig. 3e, f). It is also worth noting that AP lesions show elevated Rankl expression for osteoclast differentiation.^15^ Indeed, in the AP lesion site, *OsxCre;KL*^*fl/fl*^ mice had augmented Rankl expression compared to control mice, which was consistent at the transcription level (Fig. 3g, h). Meanwhile, *Opg* expression was reduced upon inflammation, leading to highest *Rankl/Opg* ratio in Klotho-ablated mice with AP (Fig. 3h). Bone loss is self-limiting in most cases of AP, owing to a newly established equilibrium between bone resorption and bone formation.^16^ Therefore, we next examined whether a deficiency in Klotho would affect this protective regulatory process during AP. We generated control and mutant mice that express the *tdTomato* gene in order to visualize Osx^+^ lineage cells during the progression of the infection-induced periapical bone loss. Osx-expressing cells and their progeny assessed by OsxCre-induced tdTomato expression were abundantly detected in dental pulp, periodontal ligament, as well as osteoblasts and osteocytes in alveolar bone (Fig. 3i). Immunofluorescent double labeling of tdTomato and GFP showed that AP significantly induced the generation of Osx:GFP^+^ cells, which were also tdTomato^+^ (Fig. 3j). This suggests that the pathological apical microenvironment preserves hard tissue formation capability to some degree by increasing osteogenic differentiation. More importantly, a significant reduction in Osx:GFP/tdTomato double-positive cells was detected in the mutants, implying the impaired osteogenic potential of cells that lack Klotho.

### Deletion of Klotho in Osx^+^-mesenchymal progenitors attenuates alveolar bone repair

**Fig. 4:**
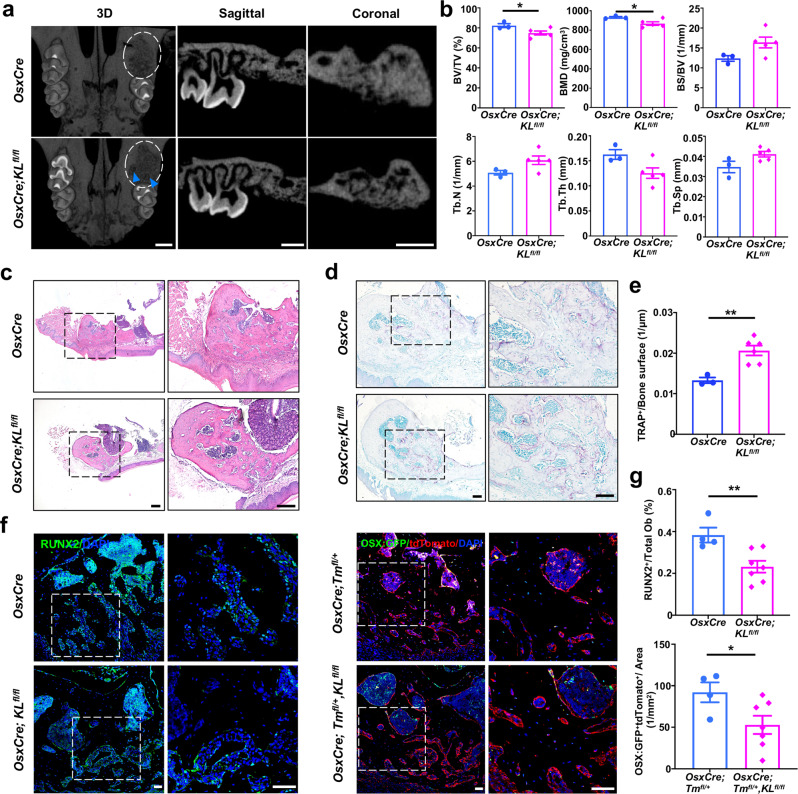
Alveolar bone repair is attenuated in *OsxCre;KL*^*fl/fl*^ mice. **a, b** μCT images and quantitative analyses of bone healing in the tooth extraction socket. White ellipses indicate extraction socket in maxilla. Blue arrowhead indicates the delayed healing of tooth socket. *n* = 3 in *OsxCre* group and *n* = 5 in *OsxCre;KL*^*fl/fl*^ group. **c–e** H&E and TRAP staining of tooth extraction socket from control and *OsxCre;KL*^*fl/fl*^ mice. Higher magnifications of boxed areas show less bone repair and more TRAP-positive cells in socket from *OsxCre;KL*^*fl/fl*^ mice. *n* = 3 in *OsxCre* group and *n* = 6 in *OsxCre;KL*^*fl/fl*^ group. **f** RUNX2 immunofluorescence staining and double fluorescence labeling of OSX:GFP and tdTomato show reduced Runx2 or OSX:GFP and tdTomato-positive osteoblasts in Klotho-deletion mice. **g** Quantification of RUNX2-positive cells or OSX:GFP and tdTomato double-positive cells in socket. *n* = 4 in *OsxCre* group and *n* = 7 in *OsxCre;KL*^*fl/fl*^ group. *p < 0.05, **p < 0.01. All data are shown as the mean ± SEM. Scale bar, 500 μm (**a**), 200 μm (**c**), 100 μm (**d**), or 50 μm (**f**)

We further assessed the function of Klotho in alveolar bone repair by generating an alveolar socket healing model. 3D volumetric μCT reconstruction revealed that bone healing at the extraction site was complete in control mice 21 days after tooth extraction. However, mutants had less organized bony surface and delayed healing of the alveolar socket (Fig. 4a). The regenerated bone occupying the healing site was then evaluated. There was a significant decrease in BV/TV and bone mineral density (BMD) in the newly formed bone of *OsxCre;KL*^*fl/fl*^ mice. No differences were observed in bone surface/bone volume (BS/BV), trabecular number (Tb. N), and Tb. Sp in mutants (Fig. 4b). *OsxCre;KL*^*fl/fl*^ mice had less interconnected alveolar bone in the extraction socket, accompanied by increased bone marrow area (Fig. 4c). Bone remodeling after tooth extraction is accomplished by the coordination of osteoblasts and osteoclasts,^17^ so we next enumerated the osteoclasts on the bone surface. There were ample osteoclasts attached to the bone surface of the healing sockets in both groups. However, the osteoclast numbers per bone perimeter increased significantly in *OsxCre;KL*^*fl/fl*^ mice (Fig. 4d, e). Immunofluorescent double labeling showed statistically reduced Runx2-positive osteoblasts in Klotho-deficient cells around the trabecular bone in the socket. Likewise, fewer GFP^+^/tdTomato^+^ osteoblasts were detected in Klotho-deficient mice versus controls (Fig. 4f, g), suggesting Klotho deletion led to suppressed osteoblast differentiation upon traumatic injury. Taken together, these results indicate that the slower-to-heal extraction sockets in *OsxCre;KL*^*fl/fl*^ mice could be due to a reduction in osteogenesis accompanied by accelerated bone resorption.

### Lack of Klotho potentiates osteoclast formation under TNF-α-induced inflammation

**Fig. 5:**
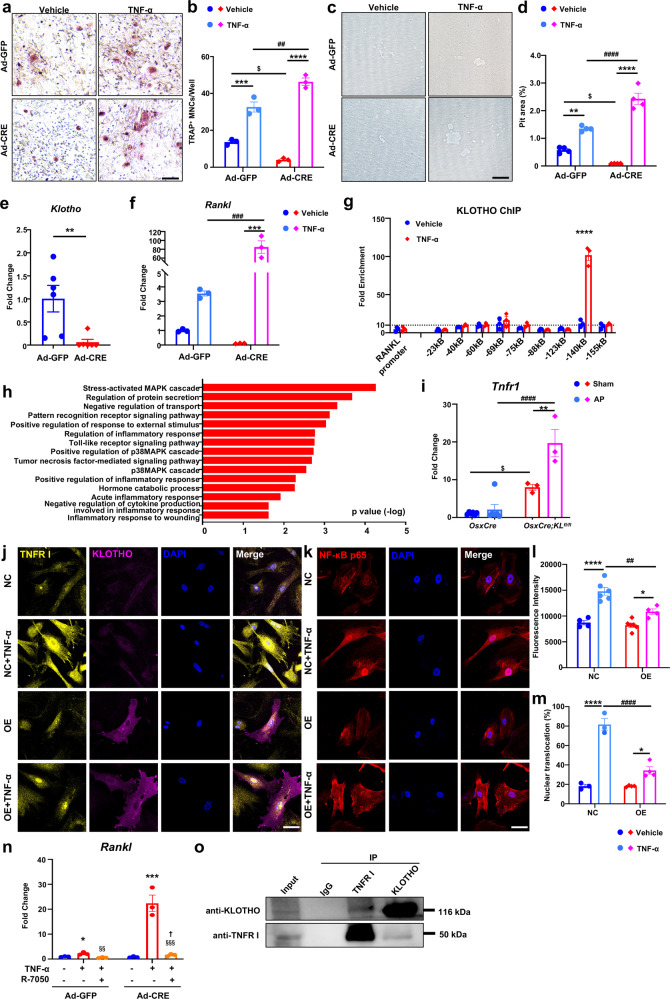
Osteoblast-Klotho mediates osteoclast formation and suppresses TNF-α-induced TNF receptor I activation. **a–d** Primary osteoblasts from *KL*^*fl/fl*^ mice were transfected with adenovirus-mediated Cre (Ad-CRE), Ad-GFP were used as control. Transfected osteoblasts and bone-marrow-derived macrophage (BMMs) co-cultured under TNF-α or vehicle treatment. **a, b** TRAP staining and quantitative analyses after 9d induction. *n* = 3. **c, d** Pit resorption assay after 13d induction. *n* = 4. **e** Gene expression of *Klotho* in osteoblasts from *KL*^*fl/fl*^ mice after adenovirus transfection. *n* = 6. **f** Transcription of osteoclastogenesis related gene *Rankl*. *n* = 3. **g** Klotho-overexpressed MC3T3 cells were treated with TNF-α (10 ng/mL, 60 min) or vehicle followed by ChIP for Klotho. DNA was quantified by qPCR and results were exhibited as fold enrichment versus control IgG. *n* = 3. **h** GO enrichment analysis of upregulated genes in DEGs. *n* = 3. **i** Gene expression of *Tnfr1* in AP model of *OsxCre;KL*^*fl/fl*^ and control mice. *n* = 6 in *OsxCre*-sham and *OsxCre*-AP groups and *n* = 3 in *OsxCre;KL*^*fl/fl*^-sham and *OsxCre;KL*^*fl/fl*^ AP groups. **j** Immunocytochemistry of Klotho and TNFR I in Klotho-overexpressed progenitors. **k** Immunocytochemistry shows NF-κB p65 distribution in cytoplasm and nucleus. **l** Quantification of TNFR I expression during TNF-α stimulation. *n* = 4 in NC-CTRL and OE (Klotho Overexpression)-TNF-α groups; *n* = 6 in NC-TNF-α and OE-CTRL groups. **m** Quantification of NF-κB p65 nuclear translocalization. *n* = 3 in NC-Vehicel and NC-TNF-α groups; *n* = 4 in OE-Vehicle and OE-TNF-α groups. **n** Gene expression of *Rankl* in primary osteoblasts and Klotho-deleted osteoblasts when treated with TNF-α in combination with R-7050 (TNFR I inhibitor). *n* = 3. **o** Coimmunoprecipitation assay was performed on Klotho-overexpressed MC3T3 cells. Proteins were collected and immunoprecipitated with Klotho or TNFR I antibodies. Western blot was performed using Klotho or TNFR I antibodies to determine the interaction between Klotho and TNFR I. IP immunoprecipitation. *, ^$^, ^†^*p* < 0.05, **, ^##^, ^§§^*p* < 0.01^, *^**,^$$$^, ^§§§^*p* < 0.001, **^**, ####^*p* < 0.0001, * indicates AP versus sham, or Vehicle versus TNF-α. ^$^ indicates *OsxCre* Sham versus *OsxCre;KL*^*fl/fl*^ Sham, or NC Vehicle versus OE Vehicle. ^#^ indicates Ad-GFP Vehicle versus Ad-CRE Vehicle, *OsxCre* AP versus *OsxCre;KL*^*fl/fl*^ AP, or NC-TNF-α versus OE-TNF-α. ^§^ indicates TNF-α versus TNF-α + R-7050. ^†^ indicate Ad-GFP TNF-α + R-7050 versus Ad-CRE TNF-α + R-7050. All data are shown as the mean ± SEM. Scale bar, 100 μm (**a**, **c**), and 50 μm (**j**, **k**)

The apical periodontitis and tooth extraction models generated for this study confirmed the adverse effect of Klotho deletion on tissue repair by mediating both osteoblast and osteoclast activity. The mechanism by which Klotho deficiency in osteoblast lineage cells mediates bone resorption was studied using calvaria-derived osteoblasts co-cultured with bone-marrow-derived osteoclasts. Klotho was ablated by administrating adenovirus-mediated Cre (Ad-CRE) to osteoblasts obtained from *KL*^*fl/fl*^ mice (Fig. 5e). Klotho-deficient osteoblasts demonstrated a reduced capability to support osteoclastogenesis, as indicated by the reduced number of TRAP^+^ multinucleated cells (Fig. 5a, b). We performed a pit resorption assay in an osteoblast–osteoclast co-culture system to determine the osteoclast activity. Consistent with the TRAP staining results, osteoclasts in the co-culture with Klotho-deficient osteoblastic cells had reduced resorption activity when compared with control osteoblasts (Fig. 5c, d). We screened for the key factors secreted by osteoblasts to tune the activity of osteoclasts and has found that *Rankl* expression was downregulated in Klotho-ablated osteoblasts while upregulated in cells with Klotho overexpression (Fig. 2e, g). Furthermore, *Rankl* were downregulated after being transfected by Ad-CRE in osteoblasts from *KL*^*fl/fl*^ mice (Fig. 5f). These data demonstrated that osteoblastic Klotho acts non-cell-autonomously to mediate osteoclast differentiation and activity.

Maxillofacial bone defects can be caused by several factors including inflammation and trauma.^2^ One of the common characteristics of inflammation and trauma-induced bone loss is the activation of TNF signaling.^18^ To explore this aspect, TNF-α was introduced into the osteoblast–osteoclast co-culture system to examine the effect of Klotho deficiency in inflammatory response and osteoclastogenesis. TNF-α amplified osteoblast-induced osteoclast differentiation in both control and mutant groups. Osteoclastogenesis was attenuated in cultured Klotho-deficient osteoblasts under normal conditions, but surprisingly there was an even more significant increase of osteoclast number and activity in co-cultures of Klotho-ablated osteoblastic cells upon TNF-α administration (Fig. 5a–d). This was accompanied by a higher *Rankl* expression indicating that Klotho may play a pivotal role in suppressing Rankl induced by TNF-α signaling in osteoblasts and subsequent osteoclast formation during inflammation (Fig. 5f). Furthermore, ChIP-qPCR was applied to detect if Klotho could bind the indicated regulatory region of *Rankl*. Treating Klotho-overexpressed osteoblasts with TNF-α revealed that Klotho inducibly associated with the −140 kB *Rankl* distal regulatory element, indicating Klotho may exhibit a nuclear function to repress *Rankl* transcription under inflammation (Fig. 5g).

### Klotho inhibits TNF-α-induced TNF receptor I activation and NF-κB signaling pathway in osteoblasts

GO analysis of genes from Klotho-depleted mandibles showed that the upregulated genes were associated with regulation of response to external stimulus, regulation of inflammatory response, and tumor necrosis factor-mediated signaling pathway (Fig. 5h). Indeed, higher expression levels of the TNF-α major receptor, TNF receptor I (*Tnfr1*), were observed in *OsxCre;KL*^*fl/fl*^ mice, with the highest level detected in *OsxCre;KL*^*fl/fl*^ mice under AP. This is a further indication that Klotho may negatively influence the inflammatory response (Fig. 5i). To test this hypothesis, we first overexpressed Klotho in osteoblasts and found significantly suppressed TNFR I expression in the cytomembrane under normal conditions (Fig. 5j). Immunofluorescence examination of control osteoblasts treated with TNF-α revealed a higher TNFR I expression compared to a vehicle-treated group. Yet this upregulation was blunted by Klotho overexpression (Fig. 5l). Furthermore, overexpression of Klotho significantly suppressed TNF-α-induced NF-κB p65 subunit nuclear translocalization, resulting in a reduction in activated osteoblasts to total osteoblast number when compared to control cells that were also subjected to TNF-α (Fig. 5k, m). R-7050 is a small molecule TNFR inhibitor that blocks TNFR I association with intracellular adaptor molecules, such as TRADD and RIP.^19^ Notably, R-7050 administration prevented TNF-α-induced *Rankl* expression and this occurred without the impact of Klotho ablation, demonstrating that Klotho affected TNFR I itself rather than its downstream molecules under TNF-α stimulation (Fig. 5n). To further investigate the possibility of an interaction between Klotho and TNFR I, coimmunoprecipitation assays were performed. The results indicated that Klotho could directly bind to TNFR I, which may interfere TNFR I signaling pathway (Fig. 5o).

### Human alveolar bone in apical periodontitis is associated with an altered Klotho expression pattern

**Fig. 6:**
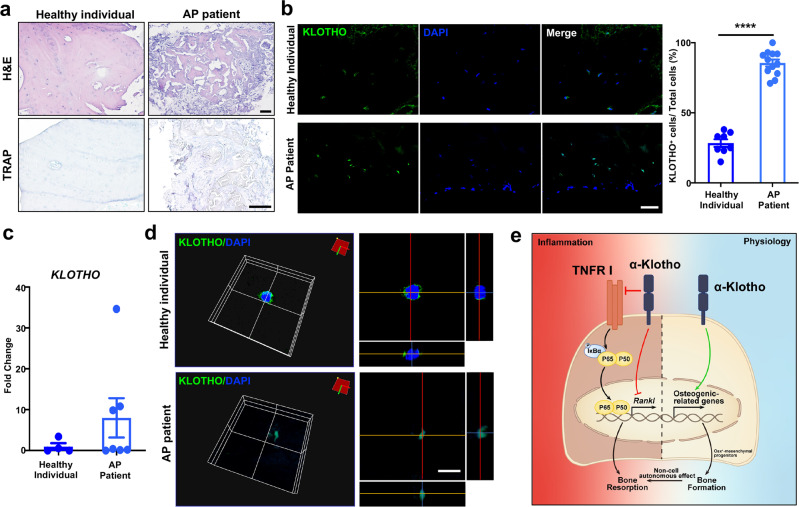
Human alveolar bone in apical periodontitis is associated with altered Klotho expression pattern. **a** H&E and TRAP staining of healthy individuals and AP patients. **b** Immunofluorescence staining and quantification of KLOTHO-positive cells in human alveolar bone. *n* = 8 in healthy individuals or 12 in AP patients. **c** Gene expression of *KLOTHO* in alveolar bone of healthy individuals and AP patients. *n* = 4 in healthy individuals and *n* = 7 in AP patients. **d** 3D images obtained from confocal microscopy show KLOTHO protein expression pattern. *n* = 4. **e** Schematic diagram showed that Klotho in Osx^+^-mensenchymal progenitors exerts pro-osteogenic and anti-inflammatory effects during mandibular bone formation and repair. All data are shown as the mean ± SEM. Scale bar, 100 μm (**a**), 50 μm (**b**), and 10 μm (**c**)

Klotho expression has been identified in a variety of human tissues, such as kidney, placenta, lung, pancreas, breast, parathyroid gland, heart, vasculature, brain, and several endocrine tissues.^20^ More recently, Klotho expression has been found in bone tissue in mice^11^ and *Klotho* gene polymorphisms have been associated with bone density changes with aging in humans.^21^ But there is a lack of detailed characterization of its expression in human bone. We examined Klotho expression in human alveolar bone tissue by collecting samples from normal individuals and patients with AP. Immunostaining demonstrated that KLOTHO was highly expressed in the cell membrane and cytoplasm of osteoblasts and osteocytes in the alveolar bone under normal physiological conditions (Fig. 6b). Under pathological states, H&E staining showed that the bone tissue was surrounded by inflammation-related cells concomitant with more TRAP-positive osteoclasts around the bone tissue (Fig. 6a). More importantly, percent of KLOTHO-positive cells was markedly elevated with AP, along with a tendency towards increased gene expression (Fig. 6b, c). The elevated *Klotho* transcription was also observed in mice with AP and tooth extraction (Supplementary Fig. 3). Intriguingly, we observed that KLOTHO protein expression was localized in the cell nucleus in AP patients, suggesting a potential nuclear function for Klotho under inflammatory conditions (Fig. 6d).

Altogether, these results demonstrate that Klotho in mensenchymal progenitors has a key role in mandibular alveolar bone formation and repair by directly promoting osteogenic differentiation and regulating osteoclastogenesis in a non-cell-autonomous manner. More importantly, osteoblastic Klotho has a central function in suppressing inflammation-induced bone loss (Fig. 6e). The distinct expression pattern of Klotho in human alveolar bone in normal physiological and pathological conditions opens up avenues for modulating Klotho expression in mensenchymal progenitors to stimulate alveolar bone formation and regeneration.

## Discussion

Here we generated a novel mouse model of Osx-specific deletion of Klotho that demonstrates a pivotal function for Klotho in the regulation of mandibular alveolar bone formation and repair. *OsxCre;KL*^*fl/fl*^ mice exhibited reduced alveolar bone formation during developmental and adult stages. Furthermore, RNA-seq analysis revealed that osteogenic-related gene expression was significantly downregulated in mutant mandibles. This suggests that Klotho expressed in Osx-expressing cells functions to stimulate osteogenesis during mandibular alveolar bone formation. The impaired osteogenic differentiation in mutants is similar to that observed in Klotho-hypomorphic mice, which exhibit reduced osteoblast numbers and osteopenia.^22^ Hikone et al. found alteration of the periodontal ligament and mandibular alveolar bone in *kl/kl* mice. The abnormalities in alveolar bone included less numbers of ALP^+^ osteoblasts and TRAP^+^ osteoclasts, which were similar to those found in *OsxCre;KL*^*fl/fl*^ mice.^23^ However, in *kl/kl* mice, the severely disturbed mineral metabolism makes it difficult to interpret to what extent the disruption of Klotho expression in bone cells accounts for the skeletal phenotype in a cell-autonomous manner. Indeed, the abnormal periodontal tissues of the interalveolar septum of *kl/kl* mice could be rescued by administrating low-Pi diet.^23^ In this study, systemic mineral ion homeostasis in *OsxCre;KL*^*fl/fl*^ mice was not affected and this allowed us to dissect the tissue-specific role of Klotho. In vitro culture of primary osteoblasts from *OsxCre;KL*^*fl/fl*^ and *OsxCre* mice further confirmed that loss of Klotho suppresses osteogenic differentiation, indicating a cell-autonomous role of Klotho in regulating alveolar bone formation. Furthermore, FGF23 expressed in osteoblasts, osteocytes, cementoblasts, and odontoblasts in mandibles, which was highly correlated with osteoblast development and matrix mineralization.^24^ The local Klotho/FGF23 signaling functions in mediating periodontal tissues and alveolar bone development.^23,24^ In our current study, gene expression of *Fgfr1* and *Egr1* in mandibles and iFGF23 were comparable between mutants and control littermates (supplementary Fig. 1d, e), suggesting Klotho may function in a FGF signaling-independed way. Moreover, ablation of Klotho results in suppressed differentiation of Osx^+^-mesenchymal progenitor cells in the repair response. The reduced osteogenic differentiation of Klotho-deficient mensenchymal progenitors seemed contradictory to the phenotype of osteocyte-specific Klotho-deletion mice, which have upregulated osteoblastic activity and bone formation rate.^11^ We hypothesize that this difference in the reported bone phenotypes in Klotho-deficient mice may be attributed to the difference in the relative impact of deletion of Klotho on specific cell types as well as the distinct characteristics of endochondral bone versus intramembranous bone formation. For example, craniofacial bone develops from migrating cranial neural crest cells, which is distinct from long bone formation.^25^ Several growth factors, receptors, and their downstream signaling pathways have diverse functions in the axial and appendicular skeleton versus craniofacial bone.^4^ The different skeletal phenotype observed in *OsxCre;KL*^*fl/fl*^ mice highlights the specific role of Klotho during mandibular alveolar bone formation. The most notable clinical example is patients with tumoral calcinosis, a homozygous missense mutation of Klotho, accompanied by severe orofacial phenotypes, such as diffuse osteopenia and patchy sclerosis in calvariae as well as shortened roots.^13^ The latter was also observed in *OsxCre;KL*^*fl/fl*^ mice under AP, highlighting the distinctive regulatory mechanisms of Klotho across different skeletal components.

Importantly we noted that Klotho deletion in Osx^+^-mesenchymal progenitors significantly depressed osteoclastogenesis. We observed a significant reduction of osteoclasts in *OsxCre;KL*^*fl/fl*^ mice, accompanied by suppressed expression of osteoclast-related markers. This phenotype is similar to that of Klotho-hypomorphic mice, which exhibited reduced osteoblast and osteoclast numbers and lower cortical bone thickness.^22,26^ Moreover, deletion of Klotho in long bone mesenchymal stem cells (*Prx1Cre;KL*^*fl/fl*^), as well as in osteocytes (*Dmp1Cre;KL*^*fl/fl*^), both showed a trend towards reduced osteoclastic resorption parameters.^11,27^

Overall alveolar bone volume in *OsxCre;KL*^*fl/fl*^ mice was relatively increased. This was due to markedly suppressed osteoclastogenesis, which outpaced the reduction in osteogenesis. This imbalance in bone formation and bone resorption ultimately led to unexpectedly high alveolar bone volume in *OsxCre;KL*^*fl/fl*^ mice. This finding is in accordance with several other observations that showed increased trabecular bone volume in *kl/kl* mice accompanied by low turnover.^28^ It should be noted that at 3 weeks of age, Klotho deletion did not significantly affect alveolar bone volume, although there was a decrease in the number of osteoblasts and osteoclasts. This could be due to the early age of the mice we analyzed, with the phenotype developing over time as the mice progressed to the adult stage.

Normal adult bone homeostasis relies on a balance between osteoblast and osteoclast activity.^29^ The primary role of osteoblasts is to deposit bone matrix, but they also synthesize and secrete paracrine molecules to coordinate with osteoclastogenesis.^30^ Osteoclast differentiation and activation from its monocytic precursor is modulated in part by a balance between Rankl and Opg secreted by osteoblasts.^31^ Consistent with our results, decreased Rankl expression has been observed in *OsxCre;KL*^*fl/fl*^ mice. Co-culture of osteoblast and osteoclast suggests that Klotho-deficient osteoblasts, accompanied by lower *Rankl* expression, failed to stimulate osteoclastogenesis in vitro. This indicates that the decreased bone resorption observed in mutants is the direct result of the functional deletion of Klotho in Osx^+^-mesenchymal progenitors. Although osteoclast-specific Klotho also promoted Rankl-induced osteoclastogenesis,^26^ *OsxCre;KL*^*fl/fl*^ mice had conditional ablation of Klotho in Osx^+^-progenitors but not in osteoclasts, suggesting that Klotho is involved in osteoblast-dependent bone resorption, at least in part, by the regulation of Rankl in osteoblasts. It has been suggested that in bone marrow mesenchymal stem cells, RANKL binding to RANK activates RANKL signaling, which negatively regulates osteoblast differentiation.^32^ We propose that the reduced bone formation in *OsxCre;KL*^*fl/fl*^ mice mainly caused by suppressed expression of other ostegenic-related factors. Rankl expression in Osx^+^-cells in *OsxCre;KL*^*fl/fl*^ mice is essential for mediating osteoclastogenesis.

Another interesting finding in this study is that bone resorption is activated in *OsxCre;KL*^*fl/fl*^ mice in conjunction with inflammatory-related bone loss and traumatic bone injury despite the repressed osteoclastogenesis seen in the normal physiological state. It is notable that osteoclast number and *Rankl/Opg* levels were higher in *Prx1Cre;KL*^*fl/fl*^ mice during uremic inflammation,^27^ indicating that Klotho in mesenchymal progentiors may mediate osteoclastogenesis under pathological conditions. Although apical periodontitis is a destructive bone pathology and tooth extraction bone healing is a regenerative process, both involve inflammation regulation.^33^ In apical periodontitis, chronic inflammation stimulates the TNF-α and the NF-κB signaling pathways, leading to osteoclast differentiation and activation of bone resorption.^34^ The tooth extraction wound-healing process consists of inflammatory, repair, and remodeling phases, where inflammation is the pivotal first step for healing.^35^ It occurs immediately after tooth extraction in response to the trauma and bacterial insults.^36^ A variety of cytokines, such as TNF-α, interleukin-1, interleukin-6, and motif chemokine 2 (CCL2), are highly expressed in the inflammatory stage.^18^ In particular, the pro-inflammatory cytokine TNF-α has a crucial role during bone resorption and bone healing. It stimulates osteoclastogenesis by enhancing Rankl production in bone marrow stromal cells, osteoblasts, and osteocytes.^37^ TNF-α activates cell signaling by binding to two types of receptors: TNFR I and TNFR II.^38^ TNFR I is thought to mediate most of the biological function of TNF-α and is responsible for Rankl synthesis in osteo-lineage cells.^39^

In this study, we found that Klotho ablation in Osx^+^-mensenchymal progenitor cells upregulated TNFR I expression concomitant with higher endogenous levels of *Tnf-α* and *Il-6* expression in mutant versus control mice. GO analysis further confirmed upregulation of inflammation pathways upon Klotho ablation. On the other hand, overexpressing Klotho in osteoblasts suppressed TNFR I expression at both the transcript and protein levels. More importantly, by direct binding of Klotho and TNFR I, Klotho diminished NF-κB nuclear translocation induced by TNF-α administration and the subsequent induction of Rankl expression, indicating the anti-inflammatory functions of Klotho in osteoblastic cells at the cellular level. This may explain the increased osteoclastogenesis observed in *OsxCre;KL*^*fl/fl*^ mice under conditions of apical periodontitis and traumatic injury. The function of Klotho under inflammation is ciritical for clinical applications since the complex microbial community in oral environment can affect the homestasis and repair of alveolar bone.^40^ *OsxCre* was used to delete Klotho in progenitor cells and thus in osteoblasts and osteocytes. Lack of Klotho in this major bone cell population caused a failure to inhibit TNFR I, which accelerated inflammation-induced Rankl production, ultimately leading to upregulated bone resorption. TNF-α also acts directly to increase differentiation of macrophages towards an osteoclast fate.^41^ We cannot exclude the possibility that the presence of these cells in vivo at the inflammatory site also contribute to the Rankl response. It would be interesting to determine whether mesenchymal progenitor-Klotho has an indirect regulatory role in immune cells in future studies.

The anti-inflammatory function of Klotho has been reported previously in other locations, such as kidney, endothelium, pancreas, and peripheral blood.^42,43^ Mice lacking Klotho exhibit enhanced inflammatory damage in animals with renal diseases.^44^ By contrast, exogenous supplementation of Klotho or Klotho transgenic expression can rescue these inflammation-associated renal pathological processes.^45^ Additionally, Klotho diminished the inflammatory response to Lipopolysaccharide in monocytes.^46^ In endothelial cells, Klotho might be associated with the inflammatory process by attenuating adhesion molecules and NF-κB expression.^43^ These observations are mainly attributed to the function of the soluble form of Klotho, generated from alternative splicing or from ectodomain shedding of membrane-bound Klotho.^47^ Taken together with our study there is an important interaction between Klotho-inflammatory response possibly mediated through negative feedback on the TNFR I. Whether the membrane-bound or soluble type of Klotho protein is dominant in this effect warrants further investigation.

Our basic understanding of the cellular expression pattern of Klotho in human tissues remains largely unexplored. Previous studies have detected both cytoplasmic and membrane-bound Klotho by immunofluorescence staining from human kidney, parathyroid gland, peripheral blood, peritoneal monocytes, cardiomyocytes, colon cancer tissues, and several human tissue-derived cells.^48^ We show here for the first time, the presence of both gene and protein expression of endogenous Klotho in human bone tissue. Its expression is restricted to cell membrane and cytoplasm in osteoblasts and osteocytes, which is in accordance with its general expression pattern. Limited studies have mentioned the nuclear function of Klotho. German et al. found abundant Klotho near the nuclear membrane in choroid plexus cells and Purkinje cells in brain.^49^ Of note, in our study, we observed that Klotho may have a nuclear function in response to external stimuli such as inflammation. Several lines of evidence support this tenet. First, the expression pattern of Klotho was dramatically altered in patients with apical periodontitis. Klotho protein shifted into the nucleus, accompanied by higher *Klotho* gene transcription both in human and mice in the presence of the disease. The upregulation of Klotho expression in alveolar bone during pathological conditions might have a protective effect by inhibiting inflammation-related bone resorption. Second, ChIP assay confirmed that Klotho was induced by TNF-α for a nuclear translocalization and bound to *Rankl* distal regulatory element. Moreover, though R-7050 blocked the stimulation of Rankl by TNF-α, a higher Rankl expression was detected in Klotho-ablated osteoblasts compared to control cells under TNF-α and R-7050 treatment, emphasizing the observed nuclear function of Klotho in suppressing Rankl. These data highlighted the potential role of Klotho beyond its previous regonized function as a membrane-bound protein.

In conclusion, we have demonstrated that specific deletion of Klotho in Osx^+^-mensenchymal progenitors decreases mandibular alveolar bone formation and bone resorption, leading to increased alveolar bone mass. We found that Klotho-deficient osteoblasts express less Rankl, which is responsible for suppressing osteoclastogenesis. Moreover, bone formation during inflammatory-related bone repair is attenuated in Klotho-deficient osteoblasts. Interestingly, Klotho is activated during alveolar bone remodeling in mice and human, where it functions to inhibit TNF-α-induced Rankl expression in osteoblast lineage cells and indirectly mediate osteoclast formation. These findings reveal novel physiological and pathological functions of mesenchymal progenitor-expressed Klotho in mediating mandibular alveolar bone development and repair.

## Materials and methods

### Ethics approval statements

All animal experiments were performed with protocols approved by the Institutional Animal Care and Use Committee at State Key Laboratory of Oral Diseases, Sichuan University. Human periapical surgical specimens were collected when all patients signed an informed consent form for participation in the study and for the use of their biological tissues. The study was reviewed and approved by the Ethics Committee of West China Hospital of Stomatology, Sichuan University.

### Animals

Floxed *Klotho* and *OsxCre* mice were described previously.^50^ *B6.Cg-Gt(ROSA)26Sortm14(CAG-tdTomato)Hze/*J mice were purchased from Jackson Laboratory. Mice with a *Klotho* conditional knockout were generated using the Cre-LoxP recombination system. *KL*^*fl/fl*^ mice were mated with *OsxCre* mice to generate *OsxCre;KL*^*fl/+*^ mice. Then, *OsxCre;KL*^*fl/+*^ male mice were interbred with *KL*^*fl/+*^ females to attain *OsxCre* (control) and *OsxCre;KL*^*fl/fl*^ (mutant) mice.

### Human periapical surgical specimens

Bone specimens from human periapical lesions were collected during endodontic surgery for use as an apical periapical (AP) group. Control bone samples were harvested from a normal region of alveolar bone during mandibular osteotomy. Samples were collected for total RNA extraction and histologic examination.

### Skeletal preparation and micro-computed tomographic (μCT) analyses

P0 mice were skinned and fixed in 95% ethanol. Alizarin Red S and Alcian blue staining were performed for analyses of cartilage and mineralized tissues. Mandibles and maxillae of 3- and 11-week-old mice were fixed in 4% paraformaldehyde overnight and then stored in 70% ethanol at 4 °C before processing. The samples were scanned using the μCT Scanner (μCT50, Scanco, Switzerland), operated at 50 kV, 200 μA, with an exposure time of 300 ms and a resolution of 7.0 μm per pixel. The images were reconstructed in three-dimensional form. Regions of interest were selected from the mandibular first molar root furcation in normal alveolar bone, 1/3 level of root apex in an apical periodontitis model and mesial root socket of the maxillary first molar in a tooth extraction model. Bone-related parameters were measured to analyze alveolar bone quality and lesion area.

### Histologic and immunologic staining

Fixed samples were decalcified in 20% EDTA (pH 7.5), embedded in paraffin and cut into 5 μm sections using an HM360 microtome (Microm). Hematoxylin (VWR) and eosin (Sigma–Aldrich) staining was performed to assess histomorphology. Tartrate-resistant acid phosphatase (TRAP) (Sigma) was used to detect osteoclasts according to the manufacturers’ protocols. For immunofluorescence staining, slides were subjected to sodium citrate buffer at 95 °C for 20 min for antigen retrieval, permeabilized with 0.5% Triton X-100 (Beyotime) for 10 min, and blocked with 5% BSA for 1 h. Slides were incubated with Anti-Runx2 (1:200, Abcam, ab23981), Anti-GFP (1:50, Santa Cruz, sc-9996), Anti-RFP (1:50, Santa Cruz, sc-390909), Anti-Klotho (1:100, R&D, AF1819), Anti-Klotho (1:100, Santa Cruz, sc-515939), or Anti-Rankl (1:100, R&D, AF462) antibody overnight at 4 °C, and a fluorescence-conjugated secondary antibody, Alexa Fluor 488 or 568 (Invitrogen, 1:1000) for 1 h at room temperature. Nuclei were counterstained with DAPI (Vector). Images were captured on an Olympus confocal microscope FV3000 (Olympus).

### RNA-seq analysis

Mandibles from control and mutant mice at postnatal day 21 (P21) were used to extract total RNA and analyze with RNA-seq. Sequencing libraries were generated using the NEBNext® UltraTM RNA Library Prep Kit for Illumina® (NEB, USA) and index codes were added to correlate sequences to each sample. The library preparations were sequenced on an Illumina Hiseq platform.

### Surgery

Eight-week-old mice were used to create apical periodontitis and tooth extraction models as described previously. Mice were anesthetized by ketamine (100 mg/kg) and xylazine (10 mg/kg). For the apical periodontitis model, the right mandibular first molar was opened using a high-speed handpiece. The root canals were probed with a #10 endodontic K-file under a stereomicroscope (Leica). The pulp chamber was exposed to the oral cavity for 3 weeks. The contralateral first molars were used as control. The tooth extraction model was created using the right maxillary first molars, which were extracted with 26 G syringe needles and forceps under the stereomicroscope. Gentle pressure was applied to stop the bleeding. Mice were fed with a soft food diet for 3 weeks after surgery and sacrificed.

### Coimmunoprecipitation assay and western blot

MC3T3 cells overexpressing Klotho were cultured in 100 mm dishes and homogenized by whole-cell lysis assay (KeyGEN). Protein concentraction was measured by enhanced BCA protein assay kit (Beyotime). Immunoprecipitation was performed by Protein A/G PLUS-Agarose Immunoprecipitation Reagent (Santa Cruz, sc-2003) according to the manufacturer’s guidelines. Then samples were degenerated at 70 °C with NuPAGE LDS Sample buffer and NuPAGE Reducing Agent (Life Technologies), separated by NuPAGE 4–12% Bis-Tris SDS/PAGE using precast gels (Invitrogen) and transferred to polyvinylidene fluoride membranes (Millipore). After 1 h blocking, the membranes were incubated with anti-KLOTHO antibody (1:1000, Cosmo Bio, KM2076) or anti-TNFR I antibody (1:1000, Abcam, ab223352) overnight. Goat anti-Rat/Rabbit IgG Secondary Antibody HRP Conjugated (1:5000, Signalway Antibody, L3012 and L35027) were used and detected by enhanced chemiluminescence kit (Bio-Rad Laboratories).

### Statistics

All data are expressed as mean ± SEM. For statistical analysis, GraphPad Prism 8.0 (GraphPad Software Inc.) was used. Two-group comparisons were evaluated by unpaired two-tailed Student’s *t*-tests, and multiple comparisons were performed by one-way analysis of variance (ANOVA) or two-way ANOVA with Bonferroni post-hoc test. *P*-values <0.05 were considered significant for all analyses.

Extended methods and information about calcein double labeling, serum measurements, cells culture, transfection, immunocytochemistry, RNA isolation, and qRT-PCR analyses, ChIP assay are described in Supplemental Materials and Methods.

## Supplementary information

Supplementary Materials

## Data Availability

The datasets for the current study are available from the corresponding author upon reasonable request.
