# Supplementary material for: Klotho in Osx+-mesenchymal progenitors exerts pro-osteogenic and anti-inflammatory effects during mandibular alveolar bone formation and repair
Source: Signal Transduct Target Ther. 2022 May 11;7:155. doi: 10.1038/s41392-022-00957-5 (PMC9090922; doi:10.1038/s41392-022-00957-5)
Supplement: Supplementary file 1 — Supplementary Materials [file 41392_2022_957_MOESM1_ESM.docx]

Supplementary Materials for

Klotho in Osx^+^-mesenchymal progenitors exerts pro-osteogenic and anti-inflammatory effects during mandibular alveolar bone formation and repair

Yi Fan^#^, Chen Cui^#^, Clifford J. Rosen, Tadatoshi Sato, Ruoshi Xu, Peiran Li, Xi Wei, Ruiye Bi, Quan Yuan^*^, Chenchen Zhou^*^

Correspondence to: chenchenzhou5510@scu.edu.cn

**This PDF file includes:**

Materials and Methods

Figures. S1 to S3

Tables S1 to S2

**Materials and Methods**

**Calcein double labeling**

To evaluate dynamic mineral apposition, mice were injected with 20 mg/kg of calcein (Sigma) at 9 and 2 days prior to sacrifice at 11 weeks old. Undecalcified mandible samples were processed to obtain 8 μm sections and parameters measured from images captured by fluorescent microscope (Olympus).

**Serum measurement**

Animals fasted 4 hours before we collected blood samples. Serum was obtained using serum separation tubes (BD Biosciences). Calcium and phosphorus of serum were measured by detection kits from Stanbio Laboratory. Serum intact FGF23 was measured with FGF-23 ELISA kit (Kainos Laboratories, Inc.).

**Cells culture and osteogenesis induction**

Primary osteoblasts were isolated from calvaria by dispase II (2 mg/mL, Roche) and collagenase type II (1 mg/mL, Worthington) digestion, and cultured in α-MEM (Gibco) supplemented with 10% fetal bovine serum (Gibco) and 1% penicillin–streptomycin (HyClone) at 37°C, 5% (v/v) CO_2_ to the third passage ^1^. The attached cells were used for experiments at passage 3 and plated at 12-well for mRNA extraction and 24-well for staining with 2 x 10^4^ cells/cm^2^. To induce osteogenesis, 10 mmol/L β-glycerophosphate and 50 μg/mL ascorbic acid were added to the culture medium and changed every 2 days. Osteoblasts were cultured in osteogenic media for 7 or 14 days and fixed with 4% paraformaldehyde. Alkaline phosphatase (ALP) staining (Beyotime) was performed at 7 days and alizarin red S staining (Sigma-Aldrich) at 14 days according to the manufacturer’s instructions. TNF-α (10 ng/mL, Sigma) was added to mimic inflammatory conditions.

For the osteoblast and osteoclast co-culture system, BMMs were isolated from the femurs and tibias and stimulated by monocyte/macrophage colony-stimulating factor (M-CSF) (50 ng/ml, Peprotech) for 3 days. Osteoblasts and BMMs were cultured together with 1, 25-dihydroxyvitamin D3 (10 nM, Sigma) and PGE2 (1 μM, Sigma). Medium was half-changed every 2 days. TRAP staining was using per instructions (Sigma). 96-well plates with a bone biomimetic synthetic surface (Osteo Assay Surface, Corning) were used to quantify pits resorption area by Image J.

**Transfection**

Adenoviral infection with CRE or green fluorescent protein (GFP) was performed as described previously ^2^. For lentiviral particles, murine Klotho cDNA was amplified and ligated into lentiviral transfer vector (pEZ-Lv233), following a standardized protocol to package and purify particles (GeneCopoeia). Osteoblasts derived from *KL^fl/fl^* mice were transfected with Ad-CRE to downregulate Klotho expression (Ad-GFP as control) or transfected with lentiviral to overexpress Klotho (Negative control particles were used as control).

**RNA extraction and quantitative real-time PCR (qRT-PCR)**

Total RNA from alveolar bone taken around the first molar from surgical and control groups and RNA from cultured osteoblasts was extracted using Trizol (Invitrogen) according to the manufacturer’s protocol. RNA samples were dissolved in RNase-free water (Beyotime) and quantified by the NanoDrop ND-1000 (Thermo Fisher Scientific). RNA was reverse-transcribed using the PrimeScript RT reagent Kit (Takara) and qRT-PCR was performed with SybrGreen Supermix (Bio-Rad Laboratories). All primers of genes used for qRT-PCR are listed in Table S1. Relative expression was calculated using a 2^ΔΔCT^ method by normalizing with *Gapdh* (glyceraldehyde-3-phosphate dehydrogenase) as the internal control.

**Chromatin immunoprecipitation (ChIP) assay**

MC3T3 cells overexpressing Klotho were stimulated by TNF-α (10 ng/mL, Sigma) for 1 h, cross-linked by 1% PFA for 10 min and then quenched with glycine. ChIP assay was perfomed by Pierce Agarose ChIP Kit (Thermo) according to the instructions. Anti-KLOTHO antibody (1 μg/IP, Cosmo Bio, KM2076) or normal rabbit IgG was incubated overnight. qRT-PCR were performed to detect the binding sites in upstream of *Rankl* promoter. Primers of ChIP assay were list in Table S2. Data were expressed as relative enrichment verses control IgG, and each experiment was performed three times.

**Immunocytochemistry**

Osteoblasts were seeded in 8-well slides and transfected with lentivirus. After TNF-α stimulation for 30 minutes, cells were fixed with paraformaldehyde, permeabilized with Triton X-100 and blocked with 5% BSA. Then slides were incubated with Anti-Klotho (1:100, R&D, AF1819), Anti-TNFR1 (1:100, Santa Cruz, sc-8436) and Anti- NF-κB p65 (1:100, Cell Signaling Technology, 8242) overnight and Alexa Fluor 568 or 647 (Invitrogen, 1:1000) for 1 hour. Nuclei were counterstained with DAPI (Vector).

**References**

1 Cui, C. *et al.* Parathyroid hormone ameliorates temporomandibular joint osteoarthritic-like changes related to age. *Cell Prolif* **53**, e12755, doi:10.1111/cpr.12755 (2020).

2 Cui, C. *et al.* Role of PTH1R Signaling in Prx1(+) Mesenchymal Progenitors during Eruption. *Journal of dental research*, 22034520934732, doi:10.1177/0022034520934732 (2020).

**
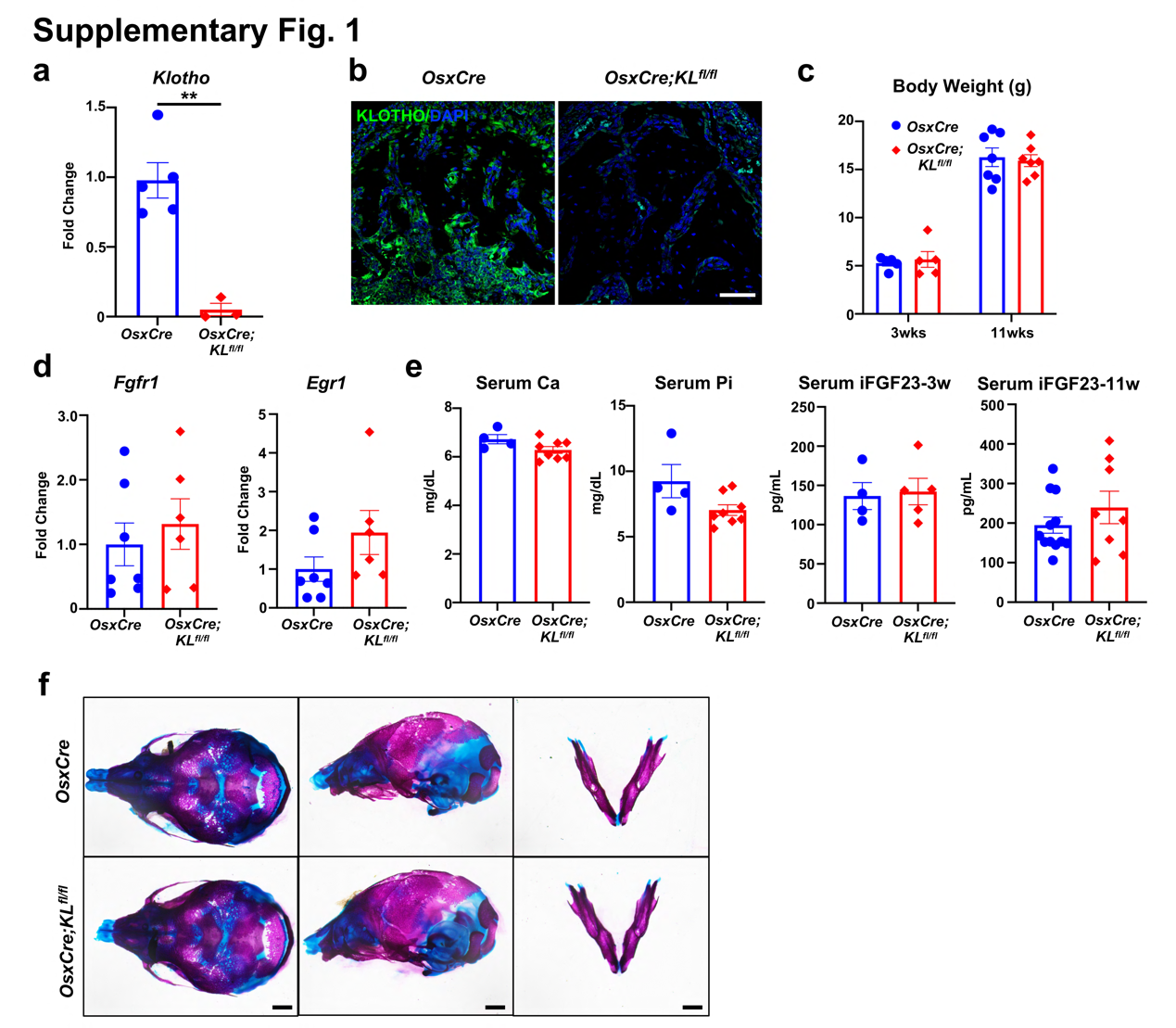
**

**Figure. S1.** Phenotype of *OsxCre;KL^fl/fl^* mice and control littermates. **a, b** Klotho expression of transcriptional (**a,** n=5 in *OsxCre* group and n=3 in *OsxCre;KL^fl/fl^* group) and translational levels (**b,** n=5) in alveolar bone. **c** Body weight of control and mutant mice at 3 and 11 weeks old. n=5 in 3-week-old and n=7 in 11-week-old mice. **d** Gene expression of *Fgfr1* and *Egr1* in mandibles. n=7 in *OsxCre* group and n=6 in *OsxCre;KL^fl/fl^* group. **e** Serum parameters of Calcium (Ca^2+^) and Phosphate (Pi) in 11-week-old mice, n=4 in *OsxCre* group and n=8 in *OsxCre;KL^fl/fl^* group; and the concentration of intact FGF23 in 3- and 11-week-old mice. n=4 in *OsxCre* group and n=5 in *OsxCre;KL^fl/fl^* group at 3-week-old, n=12 in *OsxCre* group and n=8 in *OsxCre;KL^fl/fl^* group at 11-week-old. **f** Alizarin Red/Alcian blue staining of craniofacial bone showed the distribution of cartilage and mineralization. n=4. ** *p* < 0.01. All data are shown as the mean ± SEM. Scale bar, 50 μm (**b**) and 1 mm (**f**).


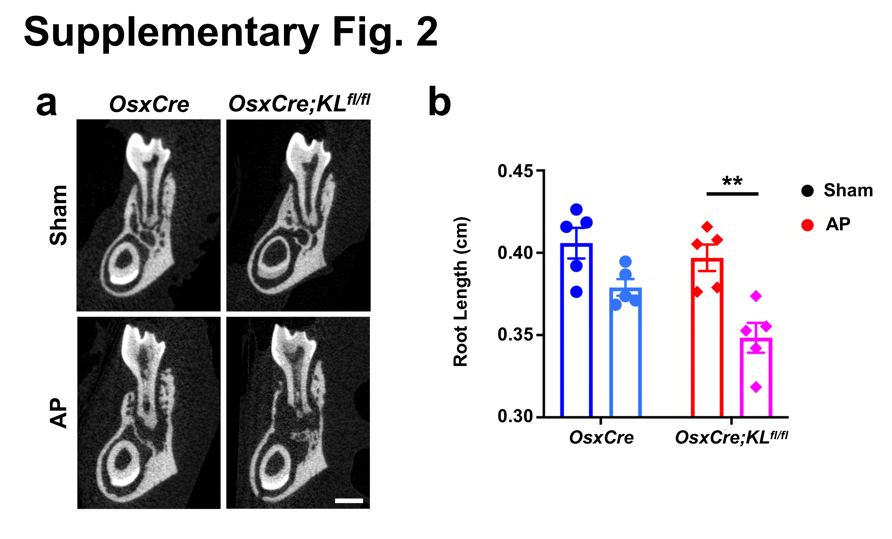


**Figure. S2.** Root length of first molar in *OsxCre* and *OsxCre;KL^fl/fl^*. **a** Coronal images of mandibular first molar were showed by μCT 3 weeks after endodontic surgery. **b** Root length were measured by ImageJ. n=5. ** *p* < 0.01. All data are shown as the mean ± SEM. Scale bar, 200 μm.


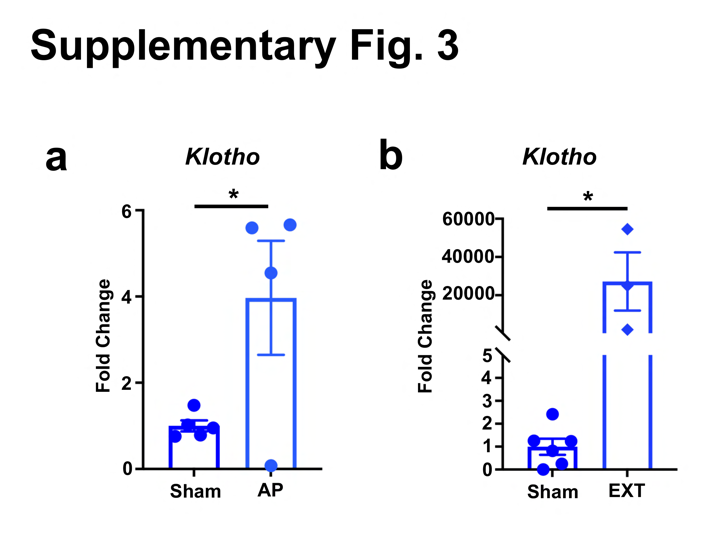


**Figure. S3.** *Klotho* gene expression in inflammatory and tooth extraction models. **a** *Klotho* expression in periapical bone of control mice and mice with AP. n=5 in *OsxCre*-sham group and n=4 in *OsxCre*-AP group. **b** *Klotho* expression in the alveolar socket at day 21 post tooth extraction (EXT). n=6 in sham group and n=3 in EXT group. * *p* < 0.05. All data are shown as the mean ± SEM.

| **Primer name** | **Forward primer** | **Reverse primer** |
| --- | --- | --- |
| mouse Acp5 | CACTCCCACCCTGAGATTTGT | CATCGTCTGCACGGTTCTG |
| mouse Mmp9 | CTGGACAGCCAGACACTAAAG | CTCGCGGCAAGTCTTCAGAG |
| mouse Rankl | GCAGAAGGAACTGCAACACA | GATGGTGAGGTGTGCAAATG |
| mouse Opg | GTTCCTGCACAGCTTCACAA | AAACAGCCCAGTGACCATTC |
| mouse Klotho | TCTCAAGAAGTTCATAATGGAAACC | CAGAAAGTCAACGTAGAAGAGTCCT |
| mouse Osx | CTCCTTGGTGGGACATGC | GTAGGCAGCTGGGGGTTC |
| mouse Runx2 | TCCACAAGGACAGAGTCAGATTACAG | CAGAAGTCAGAGGTGGCAGTGTCATC |
| mouse  Alp | CACGGCCATCCTATATGGTAA | GGGCCTGGTAGTTGTTGTGA |
| mouse Dmp1 | TGTCCTGTGCTCTCCCAGT | TTCTTCTGATGACTCACTGTTCG |
| mouse Col1α1 | GCGCTAAAGGTGCCAATG | AGCACCAGGTTCACCACTG |
| mouse Tnfr1 | CCGGGAGAAGAGGGATAGCTT | TCGGACAGTCACTCACCAAGT |
| mouse Tnf-α | CCCTCACACTCAGATCATCTTCT | GCTACGACGTGGGCTACAG |
| mouse  Il-6 | TAGTCCTTCCTACCCCAATTTCC | TTGGTCCTTAGCCACTCCTTC |
| mouse Gapdh | ACTGAGGACCAGGTTGTC | TGCTGTAGCCGTATTCATTG |
| human KLOTHO | TCCAATGGAATCGATGACG | CCATCCAGTATGTGGGCTTT |
| human GAPDH | AGCCACATCGCTCAGACAC | GCCCAATACGACCAAATCC |

**Table S1.** **qPCR primer sequences**

**Table S2.** **Primer pairs for ChIP-qPCR**

| **Primer name** | **Forward primer** | **Reverse primer** |
| --- | --- | --- |
| RANKL promoter | GGGGCCAGCCTAGAGAGCCA | GGGTACCCCAGGCAGCCCTA |
| RANKL  -23kB | CTTGGAAGGACTCCAGGAAA | CCTTTCTCAGAGCACACTGG |
| RANKL  -40kB | CACCTGTAATTCTAGCACGCA | TCACGCTCCTCTCAAATTCA |
| RANKL  -60kB | AAATCCCATTTGCTTTCCAG | GAGCTGTGTCCTAGAAGAATTGTC |
| RANKL  -69kB | TGGGAGACTCAGTTGTTGCT | TGTTGTTGGTTCGTTGTCCT |
| RANKL  -75kB | GATGGAGTCAGGATGCACAG | GAGCCCTGAGAACAGTGTGA |
| RANKL  -88kB | GAAGAGAACATTGCTGGTTGC | TAAGGATGCTTTCCCAGCTC |
| RANKL  -123kB | TGGTCCAGGTCAAGCAATAA | GGCAACACAAACCTCCTGTA |
| RANKL  -140kB | CCTCTGGGAGCAAATGAGAG | GGTGCATCTGTGGATGGTAA |
| RANKL  -155kB | CCTTGAATTCTTTGGACTGGA | TACACTGTCCTTTCCTTGCG |
